# Fluid Restriction Negatively Influences Perceived Morning Alertness and Visuomotor Ability

**DOI:** 10.3390/ijerph19010370

**Published:** 2021-12-30

**Authors:** Courteney L. Benjamin, Elliot P. Norton, Benjamin M. Shirley, Rebecca R. Rogers, Tyler D. Williams, Mallory R. Marshall, Christopher G. Ballmann

**Affiliations:** Department of Kinesiology, Samford University, Birmingham, AL 35226, USA; cbenjami@samford.edu (C.L.B.); enorton2@samford.edu (E.P.N.); bshirle1@samford.edu (B.M.S.); rrogers1@samford.edu (R.R.R.); twilli11@samford.edu (T.D.W.); mmarshal@samford.edu (M.R.M.)

**Keywords:** hypohydration, euhydration, cognition, sleep

## Abstract

The purpose of this study was to assess the effect of two fluid intake protocols on alertness and reaction time before and after fluid intake. Healthy college-age males (*n* = 12) followed two fluid intake protocols on separate occasions: (1) prescribed fluid (PF) and fluid restricted (FR). In PF, participants were instructed to consume 500 mL of fluid the night prior to and the morning of data collection. In FR, participants were instructed to refrain from the consumption of fluid for 12 h. To assess hydration status, urine specific gravity and urine color were measured. Participants perceived level of thirst and alertness were also recorded. Participants then completed visuomotor reaction time tests using the Dynavision LED board, using both a central visuomotor test and a peripheral visuomotor test (PVRT) prior to (1) and following (2) the ingestion of 100 mL of water. Participants displayed significantly improved PVRT in PF state as compared to FR (PF1 = 1.13 ± 0.16, PF2 = 1.04 ± 0.14; FR1 = 1.27 ± 0.27, FR2 = 1.18 ± 0.20; *p* = 0.038, η_p_^2^ = 0.363). Both CVRT and PVRT improved over time, following the ingestion of 100 mL of fluid. Participants in the PF state were also significantly more alert than participants in the FR state (PF = 4 ± 2, FR = 5 ± 2; *p* = 0.019, ES = 0.839). Collectively, perceived alertness and PVRT were negatively impacted by FR.

## 1. Introduction

Hydration is a critical component of nutrition and a myriad of evidence has supported that low fluid intake, or hypohydration, results in negative performance and health outcomes [1,2,3]. Although recent research has pointed to the impact of hypohydration on several domains of cognition, the impacts of mild hypohydration through relatively short bouts of fluid restriction have demonstrated mixed results [4]. One of the domains of cognitive performance is visuomotor reaction time. Visuomotor reactive ability is important for the general population for activities such as driving, as well as for athletes aiming for peak performance. Although the extent has not been fully realized in the general population, previous evidence has shown that high plasma tonicity and mild hypohydration are highly prevalent in older adults [5,6]. Thus, prevalence of mild hypohydration and the importance of reaction time in the general public and athletic populations indicates a need to further inquire as to how mild hypohydration impacts visuomotor reaction time.

Cognitive function can be separated into various domains including social cognition, intelligence, judgement, attention, memory, and executive functions [7]. Interestingly, each domain may be influenced negatively by hypohydration with either independent or synergistic decrements in cognitive ability [8]. A recent meta-analysis examining the impact of hypohydration on cognitive function classified cognitive domains into reaction time, memory, executive function, attention, information processing, and motor coordination [4,9]. The findings from this analysis demonstrated that higher level domains of cognitive function, including attention, executive function, and motor coordination, seem to be impacted by hypohydration more than lower level tasks, such as reaction time [4]. The mechanisms underpinning how hypohydration impairs higher levels of cognitive function have not been fully elucidated. One proposed mechanism for the impact of hypohydration on motor coordination is the hyperactivation of brain regions (thalamus and basal glanglia) from both perceptual sensations (such as thirst and alertness) and sensory systems responsible for motor coordination [4]. Additionally, the psychophysiological control linking perceptions of thirst and hydration status is largely under the influence of arginine vasopressin (AVP) [10]. AVP activates the thirst response in the body and has been connected to attention and mental arousal. Low AVP typically results in exacerbations to mood and alertness decrements [10]. For example, Edmonds et al. examined the impacts of fluid intake following an overnight fluid restriction, and noted improved mood and reaction time following fluid intake compared to no fluid intake [11]. Further research may grant valuable insight into the impact of hydration status on perceived thirst and alertness, as well as how perceived thirst and alertness impacts subsequent visuomotor reaction time.

Translation and comparison of findings in hydration research prove difficult as methods are heterogeneous in nature or under-reported. Methods that are commonly used to induce hypohydration include exercising, exercising in the heat, passive heat stress, fluid restriction, the use of diuretics, or a combination of these. While understanding the impact of cognitive function from active dehydration methods is aptly relevant to exercise and sport, it lacks in determining the impacts of free-living fluid restriction that naturally occurs, such as during sleep. Of the fluid restriction studies that have examined cognitive function, the length of fluid restriction ranges from 10 h to 28 h [4]. Of the few studies that have examined cognitive function following fluid restriction, only executive function and attention were shown to be significantly impacted [12,13].

While a plethora of previous literature has examined the impact of fluid restriction on various cognitive domains, methods are dissimilar and do not account for natural intraday fluctuations in thirst perception and hydration. Furthermore, little is known about the impact of short-term fluid restriction on visuomotor reaction time, considering perceived thirst and alertness levels. Therefore, the purpose of this study was to assess the effect of two fluid intake protocols on perceived thirst, alertness, and visuomotor reaction time before and after fluid intake. We hypothesized that fluid restriction will result in decreased alertness, increased levels of thirst, and increased visuomotor reaction time. We also hypothesized that fluid intake following fluid restriction will result in reduced thirst perception and improved visuomotor reaction time in the fluid restricted trial.

## 2. Materials and Methods

### 2.1. Participants

In order to determine adequate sample size, an a priori power analysis was completed using statistical software (G*power V 3.1.9.4) Since measure of alertness was the key underpinning mechanism of interest, a previous investigation using an almost identical FR period showed decreased subjective alertness with FR, which was alleviated with water ingestion (d = 1.39) was used [14]. The following parameters were used: test: *t*-test (matched pairs), d = 1.39, α = 0.05, β = 0.80. This yielded an adequate sample size of *n* = 6. In attempt to match sample sizes of previous literature [15], twelve healthy, college-aged males (age = 21 ± 1 y, height = 180.6 ± 5.3 cm, body mass = 78.1 ± 7.0 kg) were recruited from a college campus, volunteered to participate in this study and provided informed written consent. In order to participate in the study, participants had to meet the following criteria: not currently taking diuretics, normal or corrected to normal visual acuity, no prior experience with the reaction time task used in this study, not diagnosed with a concussion in the past six months, or have cardiovascular, metabolic, or renal disease. All methods and procedures were approved by the Samford University Institutional Review Board in accordance with the Declaration of Helsinki.

### 2.2. Study Design

**Figure 1:**
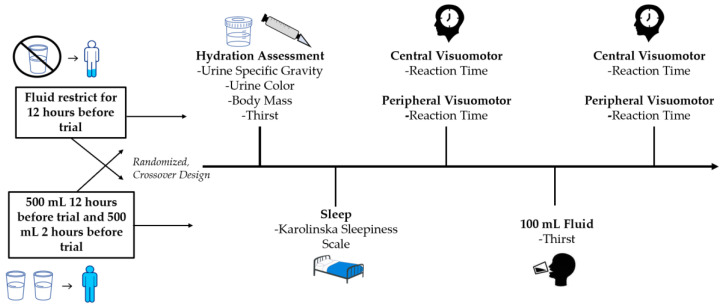
Study timeline.

An overview of the study design and protocol can be seen in Figure 1. In a randomized cross-over design, participants completed two counterbalanced trials that involved separate fluid intake protocols: prescribed fluid (PF) and restricted fluid (FR) intake. In the PF trial, participants were instructed to consume a minimum of 500 mL of water 12 h prior to the start of the trial and 500 mL of water 2 h before the start of the trial. In the FR trial, participants were instructed to consume no fluid within twelve hours of the start of the trial. For both trials, participants were instructed to refrain from strenuous physical activity for twenty-four hours and avoid caffeine, alcohol, nicotine, and pre/post workout supplements twelve hours prior to each trial. All trials were separated by at least 3 days.

### 2.3. Procedures

Upon arrival to the lab, participants provided a urine sample to assess hydration status. Urine specific gravity (USG) was assessed with a hand-held refractometer (Ketotek, Xiamen, China). Urine color was assessed using a valid urine color chart. Thirst was assessed by asking participants to rank their current level of thirst on a scale from 1–9 with anchors of “1—Not thirsty at all; 3—A little thirsty; 5—Moderatley thirsty; 7—Very thirsty; 9—Very, very thirsty” [16]. Participants were also asked to confirm their compliance of fluid intake instructions. The trial was rescheduled in the event that USG was higher than the American College of Sports Medicine threshold for euhydration (>1.020) in the PF trial or if participants indicated that they did not comply with the appropriate fluid intake protocol.

Following the hydration assessment, participants completed the Karolinska Sleepiness Scale which is on a scale from 1–10 with anchors of “1—Extremely alert; 2—Very alert; 3—Alert; 4—Rather alert; 5—Neither alert nor sleepy; 6-Some signs of sleepiness; 7—Sleepy, but no effort to keep awake; 8—Sleepy, but some effort to keep awake; 9—Very sleepy, great effort to keep awake, fighting sleep; 10-Extremely sleepy, can’t keep awake” [17]. Reaction time was then assessed using a Dynavision D2 Visuomotor board (Axtion Technology, Palatine, IL, USA). The D2 board has 64 individual lighted buttons, organized into five concentric rings and four quadrants. The D2 board is height-adjustable and was adjusted so that the tachistoscope was at eye-level for each participant [18]. The board was set in a randomized order through proactive mode A with the tachistoscope off. For all bouts, participants were instructed to focus on the center of the D2 board and use only the dominant hand to touch the lit button as fast as possible. When the participant pressed the newly lit button, the light turned off, and another button became lit, indicating a successful press [18,19].

Central visuomotor reaction time (CVRT) was assessed first with a one-minute bout that limited the parameters to the lights of rings 1 and 2, which are located towards the center of the board. Three minutes following the first CVRT test, peripheral visuomotor reaction time (PVRT) was assessed with a one-minute bout that limited the parameters to the lights of rings 3, 4, and 5, which are located towards the outside of the board. Following the first PVRT test, participants were provided with 100 mL of water. Thirst was then assessed again using the 1–9 scale, then the CVRT and PVRT tests were completed once more. The thirst assessment was completed immediately following the ingestion of fluid. The average time from which one light was pressed to the time that the following light was pressed was recorded over each one-minute testing interval as average reaction time. Peak reaction time was recorded as the shortest time from which one light was pressed to the time that the following light was pressed. Slowest reaction time was recorded as the longest time from which one light was pressed to the time that the following light was pressed. Total hits were recorded as the gross number of hits throughout each test.

### 2.4. Data Analysis

All statistical analyses were completed using Jamovi (The jamovi project (2020) Jamovi (Version1.2; Sydney, Australia). Paired samples *t*-tests were used to compare hydration indices (USG, urine color, and body mass) and perceived alertness between trials. Two-way repeated measures ANOVAs (trial: 2 levels, time: 2 levels) were used to assess thirst and reaction time parameters. Tukey correction was used for post-hoc analyses. Alpha was set at *p* < 0.05, a priori. Data are reported as mean ± standard deviation, Cohen’s d effect sizes (d) for *t*-tests and partial eta squared effect sizes (η_p_^2^) for ANOVAs. d was interpreted according to the following thresholds: <0.2 = trivial, 0.2–0.6 = small, 0.7–1.1 = moderate, 1.2–2.0 = large, and >2.0 = very large [20]. η_p_^2^ was interpreted according to the following thresholds: 0.01 = small effect, 0.06 = medium effect, 0.14 = large effect [21].

## 3. Results

**Figure 2:**
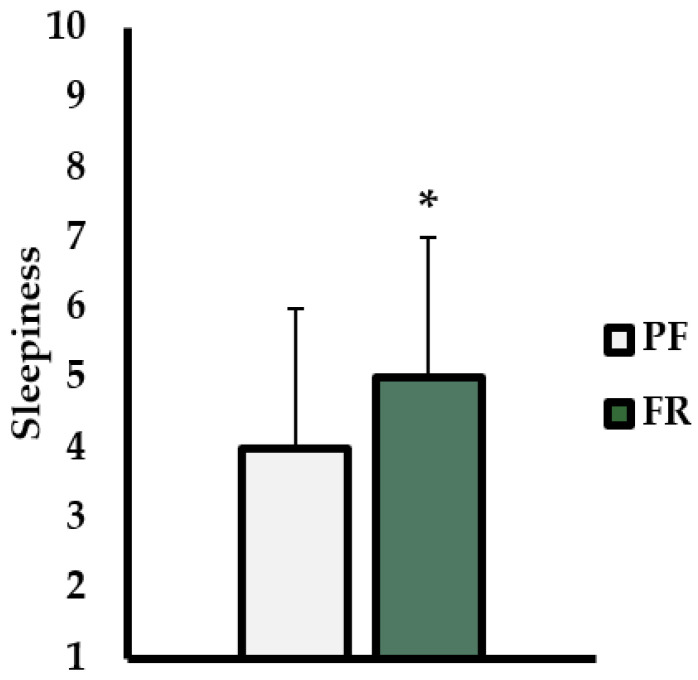
Sleep alertness values (Karolinska sleep scale). 1—Extremely alert; 2—Very alert; 3—Alert; 4—Rather alert; 5—Neither alert nor sleepy; 6—Some signs of sleepiness; 7—Sleepy, but no effort to keep awake; 8—Sleepy, but some effort to keep awake; 9—Very sleepy, great effort to keep awake, fighting sleep; 10—Extremely sleepy, can’t keep awake. Data are presented as mean ± standard deviation. * indicates statistically significant differences between trials.

USG was significantly higher in the FR trial (1.030 ± 0.007) than PF trial (1.010 ± 0.002; *p* < 0.001; d = 2.02). Urine color was significantly higher in the FR trial (6 ± 1) than the PF trial (2 ± 1; *p* < 0.001; ES = 4.39). Body mass was not different between the two trials (FR = 77.0 ± 7.8 kg; PF = 78.1 ± 7.0 kg; *p* = 0.211; ES = 0.40). The average percent body mass loss was calculated by examining differences between trials (1.7 ± 2.8 %). Thirst level prior to the start each trial (FR = 7 ± 1; PF = 3 ± 2) and following the 100 mL fluid intake were recorded FR = 5 ± 2; PF = 2 ± 2). There was a significant main effect for thirst across time (*p* < 0.001; η_p_^2^ = 0.71) and between trials (*p* < 0.001; η_p_^2^ = 0.854). There was not a significant interaction between time and trial for thirst, although it was approaching statistical significance (*p* = 0.052; η_p_^2^ = 0.33). Perceived alertness was significantly lower in the FR trial (5 ± 2) than the PF trial (4 ± 2; *p* = 0.019; ES = 0.84) (Figure 2).

CVRT data can be seen in Table 1. There was a significant main effect for time on average CVRT (*p* = 0.002) but no significant main effect for trial (*p* = 0.118). There was also no interaction between time and trial for average CVRT (*p* = 0.262). There was a significant main effect for time on CVRT for the total number of hits (*p* < 0.001) but no significant main effect for trial (*p* = 0.291). There was also no interaction between time and trial for the number of hits during the CVRT (*p* = 0.638). Post-hoc analysis demonstrated that regardless of trial, participants improved their number of hits over time (*p* < 0.001; η_p_^2^ = 0.751). There were significant main effects for time on CVRT for peak reaction time (*p* = 0.026) but no significant main effects for trial (*p* = 0.314). There was also no interaction between time and trial for the peak reaction time during the CVRT (*p* = 0.380). Post-hoc analysis demonstrated that regardless of trial, participants improved their peak reaction time over time (*p* = 0.026; η_p_^2^ = 0.407). There were no significant main effects for time (*p* = 0.783) or trial (*p* = 0.185) on CVRT for slowest reaction time. There was also no interaction between time and trial for the slowest reaction time during the CVRT (*p* = 0.335).

PVRT data can be found in Table 2. There were significant main effects for time (*p* = 0.018) and trial (*p* = 0.038); however, there was no interaction effect (*p* = 0.949). Post-hoc analysis demonstrated that average PVRT improved over time (*p* = 0.018; η_p_^2^ = 0.442) and that average PVRT was slower in the FR trial than the PR trial (*p* = 0.038; η_p_^2^ = 0.363). There were significant main effects for time on PVRT for total number of hits (*p* = 0.011) and for trial (*p* = 0.033). However, there was no interaction between time and trial for the number of hits during the PVRT (*p* = 0.558). Post-hoc analysis demonstrated that regardless of trial, participants improved their number of hits over time (*p* = 0.011; η_p_^2^ = 0.491). Post-hoc analysis also indicated that participants had a higher total number of hits in the PF trial compared to the FR trial (p = 0.033; η_p_^2^ = 0.378). There were no significant main effects for time (*p* = 0.378) or trial (*p* = 0.235) on PVRT for peak reaction time. There was also no interaction between time and trial for the peak reaction time during the PVRT (*p* = 0.651). There were no significant main effects for time (*p* = 0.230) or trial (*p* = 0.890) on PVRT for slowest reaction time. There was also no interaction between time and trial for the slowest reaction time during the PVRT (*p* = 0.143).

## 4. Discussion

The current study aimed to describe the impacts of two fluid intake protocols on perceived alertness, perceived thirst, and visuomotor reaction time. In partial agreement with our hypothesis, the findings demonstrated that FR significantly decreased perceived alertness, increased thirst perception, and worsened average PVRT. Participants demonstrated significantly improved CVRT and PVRT following the intake of 100 mL of fluid. One unexpected finding was that thirst perception was significantly improved following 100 mL of fluid intake in both trials; however, it is unknown if the improvement in CVRT and PVRT was seen due to the fluid intake or a learning effect. Findings from this study add to the literature by demonstrating the effectiveness of a PF intake protocol to significantly improve PVRT compared to FR. CVRT, however, was not impacted. These findings provide additional insight into the conflicting results of hydration on cognitive performance and reinforce previous findings of compromised reactive ability during mild hypohydration.

Previous literature examining the impacts of fluid restriction on various cognitive domains has mixed findings [4]. Weber et al. found that an approximate 12 h fast resulted in no differences in a simple reaction time task [22]. Furthermore, Watson et al. examined the impacts of 10-h FR on attention and demonstrated more errors in a cognitive task after 30 min of monotonous driving than the non-FR trial [13]. While mechanisms for differences are not fully clear, authors speculated that the increase in errors after 30 min was due to the increase in perceived task complexity over time. Differences in task complexity may bolster current findings of decrements in PVRT with FR while CVRT was largely unaffected. Specifically, peripheral vision seems to play a larger role in perception and the control of movement than central vision, potentially making PVRT a more complex task than CVRT [23]. Furthermore, previous work has shown that maintenance central vision is generally prioritized over peripheral [24,25].

Although partially dependent on the skill of the performer, previous evidence explicated that prioritization of central visual field limits the useful view of peripheral vision during complex tasks [26]. While speculative, decrements in PVRT may have currently been more pronounced with hypohydration versus CVRT due to the already narrowing of useful PVRT with vision prioritization and task complexity. Indeed, complexity in the task may result in decrements in cognitive performance following FR due to an overwhelming of the regions of the brain responsible for both motor coordination and perceptual responses [4,26]. Additionally, the frontal lobe, responsible for attention and executive function tasks, has been shown to respond to experimentally induced thirst [27].

In the current study, participants reported feeling ‘very thirsty’ in the FR trial compared to ‘a little thirsty’ in the PF trial. Additionally, participants on average felt ‘neither alert nor sleepy’ in the FR trial while they felt ‘rather alert’ in the PF trial. These findings are in agreement with a previous study that investigated cognitive–motor performance following a 24-h FR protocol that found that tiredness, alertness, and effort were worse compared to no FR [28]. Current findings may also manifest themselves in differences in alertness between PF and RF trials. Ryu et al. showed deformation of useful visual field is most pronounced in PVRT with decreased alertness and complexity of task [26]. While not fully confirmed, present impairments in PVRT with hypohydration may have been mediated by lower psychological arousal induced by FR. While purely speculative, previous investigations have reported lower cAMP responsive element binding (CREB), a marker of neural activation, to be markedly decreased in hippocampal cells that are dehydrated which may indicate changes in neuronal activity [29]. As the hippocampus and reticular formations of the brain aid in control of alertness, the mild hypohydration found presently may have diminished activation of portions of the brain important for optimizing arousal and reactive ability which peripheral fields may be particularly sensitive to changes. Future studies employing imaging techniques (i.e., fMRI, PET Scan, etc.) will be necessary to confirm proposed mechanisms.

While the authors attempted to reduce extraneous variables, this study was not without error. Both groups in the current study displayed significantly decreased thirst sensation following the intake of 100 mL of fluid when compared to thirst upon arrival. The fluid prescription described in this study was effective in significantly reducing the thirst sensation. One unexpected finding from this study was that thirst sensation decreased in both the FR and PF groups following the fluid prescription. Specifically, thirst sensation moved from ‘very thirsty’ to ‘moderately thirsty’ in the FR group and from ‘a little thirsty’ to between ‘a little thirsty’ and ‘not thirsty at all’ in the PF group. Importantly, the water prescription accomplished this while not altering overall hydration status since the volume was so small [27]. Participants in both groups significantly improved their average reaction time and total number of hits from the first to second-time point for both CVRT and PVRT. This finding could indicate that the fluid intake led to oropharyngeal stimulation, decreasing AVP, and ultimately, lowering thirst sensation [28]. This improved thirst sensation could have resulted in improved reaction times [10]. However, another possible reason for this finding is participants’ increasing familiarity with the test itself, although the counterbalanced nature of the study design should have accounted for some of the learning effects. Future research should examine this question further. A one-item alertness scale showed significant differences between the trials; however, future research should consider collecting this information for more than one day, if possible.

## 5. Conclusions

In conclusion, the present investigation provides novel information regarding the effects of FR and PF protocols on thirst sensation, perceived alertness, and PVRT. Findings from this study point to negative consequences of a relatively short fluid restriction protocol. While previous research has not demonstrated a major impact on hypohydration on reaction time, the findings from this study show that components of reaction time (involving the peripheral field of vision) are negatively impacted by mild hypohydration. Potential mechanisms for this finding include lower levels of perceived alertness and increased thirst; however, future studies are needed to determine the detailed underlying mechanisms. Future investigations should further examine the underlying physiological mechanisms responsible for the noted changes of PVRT.

## Figures and Tables

**Table 1:** Central visuomotor reaction time tests. Data are presented as mean (m) ± standard deviation (sd).

	Prescribed Fluid		Fluid Restricted	
	Test 1	Test 2	Test 1	Test 2
**Average** (s)	0.58 ± 0.05	0.57 ± 0.06 *	0.61 ± 0.04	0.59 ± 0.05 *
**Peak** (s)	0.38 ± 0.03	0.38 ± 0.04 *	0.41 ± 0.03	0.39 ± 0.03 *
**Slowest** (s)	1.27 ± 0.27	1.30 ± 0.27	1.24 ± 0.13	1.19 ± 0.20
**Total Hits** (count)	103 ± 8	106 ± 10 *	98 ± 7	102 ± 8 *

* indicates statistically significant differences from test 1 to test 2.

**Table 2:** Peripheral visuomotor reaction time tests. Data are presented as mean (m) ± standard deviation (sd).

	Prescribed Fluid		Fluid Restricted	
	Test 1	Test 2	Test 1	Test 2
**Average** (s)	1.13 ± 0.16	1.04 ± 0.14 *	1.27 ± 0.27 ^+^	1.18 ± 0.20 *^,^^+^
**Peak** (s)	0.58 ± 0.08	0.56 ± 0.06	0.60 ± 0.09	0.60 ± 0.11
**Slowest** (s)	3.57 ± 1.41	2.70 ± 0.68	3.02 ± 0.69	3.14 ± 1.09
**Total Hits** (count)	51 ± 8	56 ± 8 *	47 ± 10 ^+^	51 ± 9 *^,^^+^

* indicates statistically significant differences from test 1 to test 2. ^+^ indicates statistically significant differences between groups.

## Data Availability

Data are contained and available within this manuscript.
